# JNK1 is not essential for TNF-mediated joint disease

**DOI:** 10.1186/ar1473

**Published:** 2004-12-07

**Authors:** Marcus Köller, Silvia Hayer, Kurt Redlich, Romeo Ricci, Jean-Pierre David, Günter Steiner, Josef S Smolen, Erwin F Wagner, Georg Schett

**Affiliations:** 1 Department of Internal Medicine III, Division of Rheumatology, Medical University of Vienna, Austria; 2 CeMM, Center of Molecular Medicine of the Austrian Academy of Sciences, Vienna, Austria; 3 Research Institute of Molecular Pathology, Vienna, Austria

**Keywords:** arthritis, JNK1, TNF-α transgenic

## Abstract

Tumour necrosis factor (TNF) signalling molecules are considered as promising therapeutic targets of antirheumatic therapy. Among them, mitogen-activated protein kinases are thought to be of central importance. Herein, we investigate the role *in vivo *of TNF-α signalling through c-Jun N-terminal kinase (JNK)1 in destructive arthritis. Human TNF transgenic (hTNFtg) mice, which develop inflammatory arthritis, were intercrossed with JNK1-deficient (*JNK1*^-/-^) mice. Animals (*n *= 35) of all four genotypes (wild-type, *JNK1*^-/-^, hTNFtg, *JNK1*^-/-^hTNFtg) were assessed for clinical and histological signs of arthritis. Clinical features of arthritis (swelling and decreased grip strength) developed equally in hTNFtg and *JNK1*^-/-^hTNFtg mice. Histological analyses revealed no differences in the quantity of synovial inflammation and bone erosions or in the cellular composition of the synovial infiltrate. Bone destruction and osteoclast formation were observed to a similar degree in hTNFtg and *JNK1*^-/-^hTNFtg animals. Moreover, cartilage damage, as indicated by proteoglycan loss in the articular cartilage, was comparable in the two strains. Intact phosphorylation of JNK and c-Jun as well as expression of JNK2 in the synovial tissue of *JNK1*^-/-^hTNFtg mice suggests that signalling through JNK2 may compensate for the deficiency in JNK1. Thus, JNK1 activation does not seem to be essential for TNF-mediated arthritis.

## Introduction

Proinflammatory cytokines bind to their receptors on the plasma membrane and transmit the stimulatory effects to the nucleus via intracellular signalling molecules. Therefore, these cytokines are considered as promising therapeutic targets. Drugs specifically inhibiting such proteins are usually small molecules and are thought to open a new frontier in antirheumatic therapy along with newly arisen cytokine-blocking strategies. Among the many downstream molecules of cytokine signalling, mitogen-activated protein kinases (MAPKs) are of central importance in shuttling the signal of proinflammatory cytokines, such as IL-1 or tumour necrosis factor (TNF)-α, to their respective target tissues [1,2].

Cellular activation by TNF-α is a critical step in chronic synovial inflammation and progressive joint destruction. This is supported by the overwhelming effects of TNF blockade, which has revolutionized the therapy of rheumatoid arthritis (RA). It inhibits both inflammation and destruction of joints, in a majority of patients suffering from RA [3-5]. The hypothesis is supported by animal models in which specific overexpression of TNF-α is sufficient to cause chronic destructive arthritis [6]. Increased levels of this cytokine in the synovial fluid and tissue of RA patients have also been reported [7-9]).).

To design therapeutic tools that not only interfere with TNF signalling but also effectively block TNF-mediated inflammatory responses, it is essential to identify the major signalling targets of TNF in inflammatory joint disease. In fact, TNF-α signalling is a complex process, involving not only MAPKs but also other pathways including nuclear factor κB and the caspase cascade [10,11]. MAPKs are thought to be of central importance for mediating the proinflammatory effects of TNF-α. Interestingly, all three MAPK families – p38 protein kinase, extracellular signal-regulated kinase, and c-Jun N-terminal kinase (JNK) – are activated in the synovial membrane of RA patients, and TNF-α has the potential to signal through all of them [12]. Therefore, each of these different MAPKs is a possible therapeutic target.

We investigated the role of JNK1 in TNF-mediated inflammatory joint disease. Our findings show that the JNK1 signal pathway is not essential for the development of arthritis and joint destruction.

## Materials and methods

### Animals

The heterozygous human TNF transgenic (hTNFtg) mouse (strain: tg197; background: C57/BL6) has been described previously [6]. As reported elsewhere, mice of this strain develop destructive arthritis resembling RA within 4–6 weeks of birth [6,13]. JNK1-deficient (*JNK1*^-/-^) mice were generated as previously described [14]. The hTNFtg and *JNK1*^-/- ^strains were intercrossed to obtain double mutant animals. F_2 _generations were used and all data were generated from littermates. A total of 35 mice (wt, *n *= 7; hTNFtg, *n *= 13; JNK1^-/-^, *n *= 6; and JNK1^-/-^hTNFtg, *n *= 9) of six different breedings were studied. This study was approved by the local ethical committee pf the Medical University of Vienna.

### Clinical assessment

Arthritis was evaluated in a blinded manner as described in earlier reports [13]. Assessments were started when the mice were 5 weeks old and were repeated weekly. In brief, joint swelling was assessed using a clinical score graded from 0 to 3 (0, no swelling; 1, mild swelling of toes and ankle; 2, moderate swelling of toes and ankle; 3, severe swelling of toes and ankle). In addition, the grip strength of each paw was analysed on a wire 3 mm in diameter, using a score from 0 to -4 (0, normal grip strength; -1, mildly reduced grip strength; -2, moderately reduced grip strength; -3, severely reduced grip strength; -4, no grip strength at all). After cervical dislocation, the blood was withdrawn by heart puncture and the paws of all animals were dissected and preserved for histological analysis. The last evaluation was performed 10 weeks after birth.

### Histological sections and histochemistry

A total of 26 mice (wt, *n *= 7; hTNFtg, *n *= 6; *JNK1*^-/-^, *n *= 6; and *JNK1*^-/-^hTNFtg, *n *= 7) were assessed histologically. Hind and front paws and right knee joints were fixed in 4.0% formalin overnight and then were decalcified in a 14% EDTA/ammonium hydroxide buffer at pH 7.2 (Sigma-Aldrich, St Louis, MO, USA) at 4°C until the bones were pliable. Serial paraffin sections (2 μm) were stained with H&E, or with toluidine blue for tartrate-resistant acid phosphatase (TRAP) activity. TRAP staining was performed as previously described [13]. For immunohistochemistry, deparaffinized, ethanol-dehydrated tissue sections were boiled for 2 min in 10 mM sodium citrate buffer (pH 6.0) using a 700-W microwave oven. Tissue sections were cooled to room temperature and then rinsed using detergent solution: 0.5% Tween in phosphate-buffered saline (PBS).

For quantification of inflammation, areas of H-&-E-stained sections were measured (5 sections/animal). The total area of inflammation for each single animal was calculated by evaluating all digital, carpal, and tarsal joints and the right knee joint. The same H-&-E-stained sections were analysed as described above for quantification of erosions. The number of osteoclasts was counted as described above analysing TRAP-stained serial sections. Cartilage breakdown (i.e., proteoglycan loss and matrix dissolution) was measured from toluidine-blue-stained serial sections by assessing cartilage according to the method of Joosten and colleagues [15]. Total and destained cartilage areas were measured and percentages of destained areas indicating low proteoglycan content were ascertained.

For immunohistochemistry, dewaxed, ethanol-dehydrated tissue sections were boiled for 2 min in 10 mM sodium citrate buffer (pH 6.0) using a 700-W microwave oven, then allowed to cool to room temperature and rinsed in detergent solution (0.5% Tween in PBS) for 10 min. Tissue sections were blocked for 20 min in PBS containing 20% rabbit serum and were then incubated for 1 hour at room temperature with the following antibodies (Abs): rat monoclonal antimacrophage (F4/80) Ab (Serotec Inc, Oxford, UK); diluted 1:80), rat monoclonal anti-CD3 Ab (Novocastra, Newcastle, UK; diluted 1:100), rat monoclonal anti-CD45R/B220 Ab (Pharmingen International, Oxford, UK) and rat monoclonal antineutrophil Ab (clone7/4, Serotec), mouse monoclonal antiphosphorylated-JNK Ab (clone G7, Santa Cruz Biotechnology, Santa Cruz, CA, USA), mouse monoclonal anti-JNK2 Ab (clone D2, Santa Cruz), and mouse monoclonal phospho-specific anti-c-Jun Ab (clone KM-1, Santa Cruz). The sections were rinsed, and then endogenous peroxidase was blocked with 0.3% hydrogen peroxide in Tris-buffered saline (10 mM Tris/HCl, 140 mM NaCl, pH 7.4) for 10 min. This was followed by 30 min of incubation with a biotinylated antirat IgG secondary Ab (Vector, Burlingame, CA, USA). Then, sections were incubated with the appropriate VECTASTAIN@ABC reagent (Vector) for another 30 min using 3,3'-diaminobenzidine (Sigma).

### Statistical analysis

Data are shown as means ± standard deviation. Group mean values were compared using a two-tailed Student's *t *test.

## Results

### TNF-induced clinical signs of arthritis develop independently of JNK1

**Figure 1:**
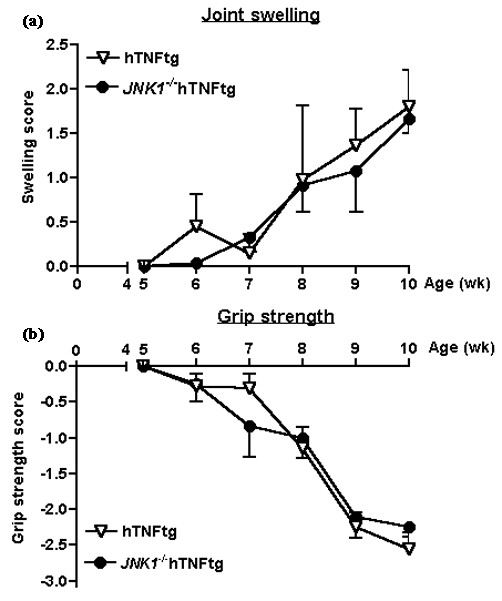
Clinical course of arthritis in human tumour necrosis factor transgenic (hTNFtg) and *JNK1*^-/-^hTNFtg mice. Joint swelling **(a) **and grip strength **(b) **were assessed. No statistically significant differences between the two groups were detected. Vertical bars indicate standard deviation. Control animals (wild-type and *JNK1*^-/-^) showed no signs of arthritis (not shown). JNK, c-Jun N-terminal kinase.

To study the role *in vivo *of JNK1 activation in TNF-mediated arthritis, we intercrossed hTNFtg with *JNK1*^-/- ^mice. The offspring of all four genotypes (wt, hTNFtg, *JNK1*^-/-^, and *JNK1*^-/-^hTNFtg) were born at Mendelian frequency and were viable. To evaluate arthritis, we assessed joint swelling and grip strength weekly in all four genotypes. Neither wt nor *JNK1*^-/- ^mice developed any signs of paw swelling, and both maintained normal grip strength (data not shown). In contrast, the hTNFtg animals developed joint swelling at 6 weeks of age, which increased to a maximum at week 10 (*P *< 0.05 in comparison with wt and *JNK1*^-/-^). In the *JNK1*^-/-^hTNFtg group, joint swelling started at age 7 weeks and increased significantly thereafter (*P *< 0.05 in comparison with wt and *JNK1*^-/-^). There was no significant difference between the hTNFtg and *JNK1*^-/-^hTNFtg mice at any time of the analysis (Fig. 1a). In addition, grip strength significantly decreased in both hTNFtg and *JNK1*^-/-^hTNFtg strains, but no significant difference between the two genotypes was found (Fig. 1b). Thus, overexpression of TNF-α induces clinical signs of arthritis also in the absence of JNK1.

### Similar degrees of synovial inflammation and cellular composition of synovial inflammatory tissue in hTNFtg and *JNK1*^-/-^hTNFtg mice

**Figure 2:**
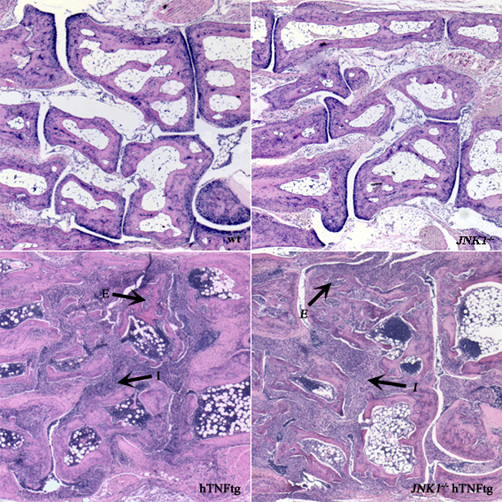
Histological assessment of synovial inflammation and joint destruction. No signs of arthritis are seen in the tarsal joints of wild-type (wt) and JNK1 (c-Jun N-terminal kinase 1) knockout (*JNK1*^-/-^) mice, whereas severe inflammation (I) and numerous erosions (E) are observed in human TNF transgenic (hTNFtg) and intercrossed (*JNK1*^-/-^hTNFtg) mice. H-&-E-stained sections; magnification 50×.

**Figure 3:**
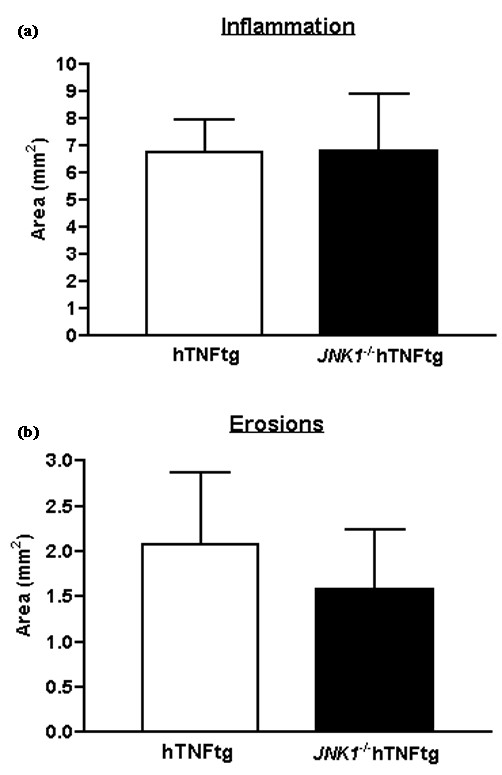
Quantitative analysis of synovial inflammation and joint destruction. There were no differences in the extent of inflammatory tissue between human tumour necrosis factor transgenic (hTNFtg) and intercrossed (*JNK1*^-/-^hTNFtg) mice. Mean areas of inflammation **(a) **and erosions **(b) **were comparable in these two strains. Vertical bars indicate standard deviation. JNK, c-Jun N-terminal kinase.

We next more closely evaluated arthritis by quantitative and qualitative histological analysis of inflammatory tissue (Fig. 2). Animals of both control groups, wt and *JNK1*^-/-^, did not show any sign of joint inflammation or destruction. In contrast, hTNFtg mice not only developed intense inflammation but also showed multiple bone erosions. Comparable destructive changes were observed in joints from *JNK1*^-/-^hTNFtg mice. Quantitative analysis of the area of synovial inflammation revealed no significant differences between hTNFtg and *JNK1*^-/-^hTNFtg mice (Fig. 3a). Similarly, quantification of erosive changes was comparable in these two genotypes (Fig. 3b). Furthermore, immunohistochemical analysis revealed similar distributions of T cells, B cells, granulocytes, and macrophages within the synovial membranes of the two genotypes (Table 1).

### Synovial osteoclast formation is not affected by the absence of JNK1

**Figure 4:**
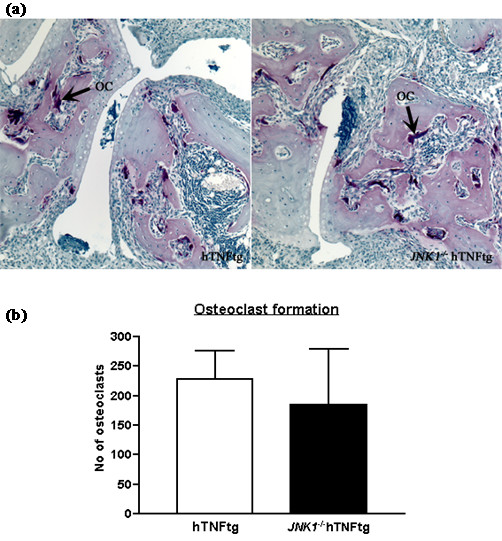
Synovial osteoclasts in human tumour necrosis factor transgenic (hTNFtg) and *JNK1*^-/-^hTNFtg mice. As demonstrated by staining with tartrate-resistant acid phosphatase **(a)**, there are abundant osteoclasts (OC) at the site of erosions in hTNFtg and intercrossed (*JNK1*^-/-^hTNFtg) mice, which all developed arthritis (magnification 100×). The number of osteoclasts was similar in the two animal groups **(b)**. Vertical bars indicate standard deviation. JNK, c-Jun N-terminal kinase.

Since JNK1 is involved in osteoclast differentiation, we wanted to know whether the absence of JNK1 influences TNF-driven osteoclast formation in the inflamed joints. Staining for the osteoclast-specific enzyme TRAP revealed numerous osteoclasts within arthritic bone erosions in both hTNFtg and *JNK1*^-/-^hTNFtg mice (Fig. 4a). Quantification of osteoclasts revealed no significant difference between the two groups (Fig. 4b), reflecting that the absence of JNK1 does not alter the progression of joint destruction in conditions of TNF overexpression.

### Proteoglycan content is reduced in cartilage of hTNFtg and *JNK1*^-/-^/hTNFtg mice

**Figure 5:**
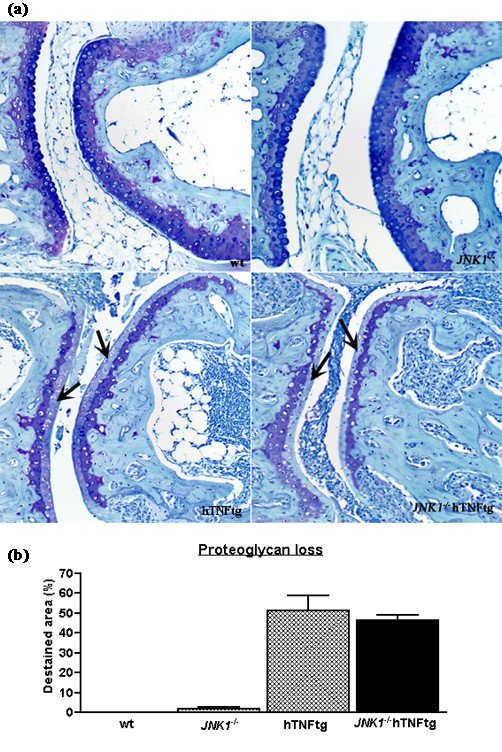
Evaluation of proteoglycan loss in human tumour necrosis factor transgenic (hTNFtg) and *JNK1*^-/-^hTNFtg mice. As shown by toluidine blue staining **(a)**, arthritis leads to loss of proteoglycan (arrows) in the cartilage of hTNFtg and intercrossed *JNK1*^-/-^hTNFtg mice (magnification 100×). The area of early cartilage damage **(b) **was similar in the two groups with arthritis, whereas the joints of wild-type (wt) and JNK1 knockout (*JNK1*^-/-^) animals were unaffected. Vertical bars indicate standard deviation. JNK, c-Jun N-terminal kinase.

Lastly, we addressed whether the absence of JNK1 influences cartilage damage in TNF-α-induced arthritis. Quantitative assessment of proteoglycan loss by toluidine blue staining showed a marked reduction of proteoglycan content in both hTNFtg and *JNK1*^-/-^hTNFtg mice (Fig. 5a). But again, no significant difference in the percentage of the affected cartilage surface was detected between hTNFtg and *JNK1*^-/-^hTNFtg mice. In contrast, wt and *JNK1*^-/- ^animals revealed virtually no alterations of articular cartilage (Fig. 5b). This suggests that TNF-α induces degradation of cartilage independently of JNK1.

### Lack of JNK1 decreases expression of phosphorylated JNK but does not affect activation of c-Jun

**Figure 6:**
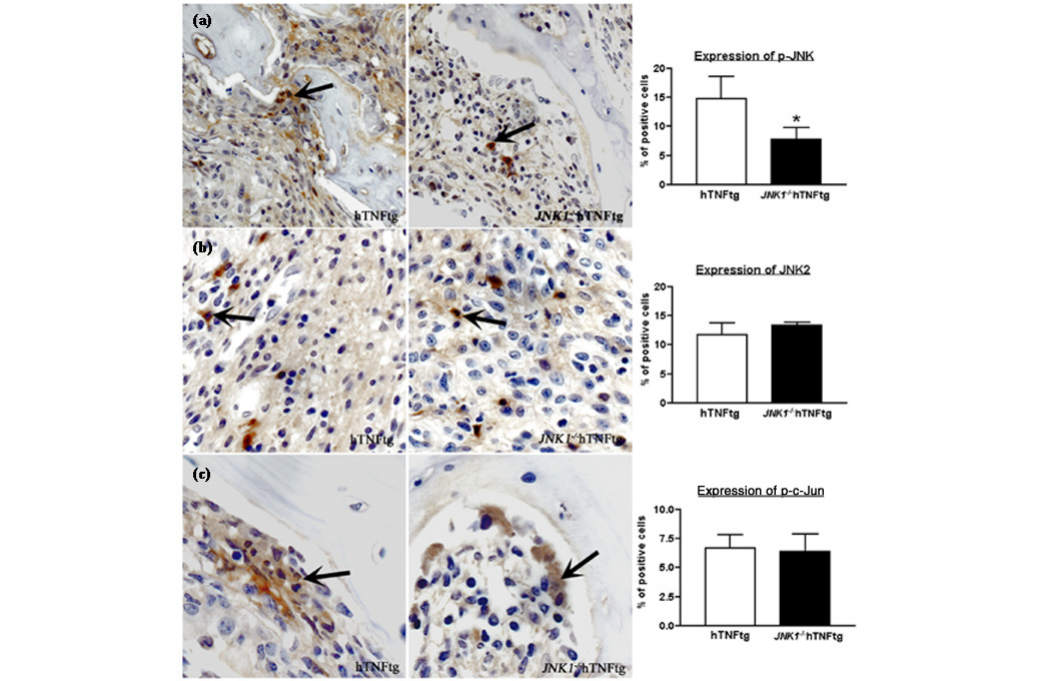
Analysis of JNK activation in human tumour necrosis factor transgenic (hTNFtg) and *JNK1*^-/-^/hTNFtg mice. Synovial inflammatory tissue of the mice were stained with antibodies against the phosphorylated form of JNK **(a)**, total JNK2 **(b)**, and the phosphorylated forms of c-Jun **(c)**. Positive cells are stained in brown (arrows; magnification 400×). Results of quantification (percentages of positive cells) are given in separate bar charts. Vertical bars indicate standard deviation. JNK, c-Jun N-terminal kinase; p-c-Jun, phosphorylated c-Jun; p-JNK, phosphorylated JNK. 1 cm = (a) 40 μm, (b, c) 30 μm.

Surprisingly, *JNK1*^-/-^hTNFtg mice developed destructive arthritis comparable to that of hTNFtg animals. Therefore, we assessed JNK signalling in animals of both genotypes. Histological staining of synovial membrane from *JNK1*^-/- ^hTNFtg mice revealed significantly fewer cells expressing phosphorylated JNK than in hTNFtg animals (Fig. 6a; *P *< 0.05). The expression within the synovial membrane of JNK2 (Fig. 6b) or phosphorylated c-Jun (Fig. 6c) was similar in both groups that developed arthritis.

## Discussion

In RA, cytokine-mediated cell activation leads to chronic synovial inflammation as well as bone and cartilage destruction. Evidence from basic and clinical research has established TNF-α as one of the major players in the pathogenesis of RA. The effects of TNF-α are mediated via membrane receptors which themselves activate intracellular messenger cascades, such as MAPK. JNKs are especially important, because of their ability to phosphorylate the activator protein (AP)-1 component c-Jun, making them critical regulators of transcription [16]. The JNK proteins include three different isoforms, of which JNK1 and (with even higher affinity) JNK2 phosphorylate c-Jun [17]. JNK2 is preferentially bound to c-Jun in unstimulated cells, whereas JNK1 becomes the major c-Jun-interacting kinase after cell stimulation [18]. Notably, JNK1 appears to be a key regulator of the differentiation of type 1 T helper cells in mice [19].

Like other MAPK pathways, JNK signalling is activated in the synovial tissue of RA patients [12,20-22]. Studies with cultured synovial fibroblast-like cells from RA patients have established proinflammatory cytokines, such as TNF-α and IL-1, as important activators of JNK signalling, and JNK2 is the dominant isoform in synovial cells [12,20-22]. In these cells, TNF-induced phosphorylation of JNK leads to the phosphorylation of c-Jun and finally activation of the transcription factor complex AP-1 [20]. Loss of either JNK1 or JNK2 suppresses AP-1 [21]. The JNK pathway is therefore involved in the regulation of genes coding for collagenases, chemoattractants such as macrophage chemoattractant protein-1, or the adhesion molecule E-selectin [23,24].

These data indicate that JNK1 is dispensable in TNF-mediated joint disease. Of interest, arthritis in hTNFtg mice is directly induced by overexpression of TNF, a potent trigger of the JNK pathway. Thus, it is surprising that even if disease is caused by the overexpression of a trigger of JNK-signalling, the absence of JNK1 is not essential for the development of the disease. However, the hTNFtg animal model has its limits, since it bypasses the autoimmune inductive phase, which is seen in other arthritis models such as collagen-induced arthritis or adjuvant arthritis, as well as in human disease. Thus, although these findings are relevant for TNF-mediated inflammation and connective tissue destruction, their implication for human RA should be seen with caution. Interestingly, the role of JNK has been investigated in autoimmune-based models of experimental arthritis in two outstanding studies. Treatment of rat adjuvant arthritis with SP600125, an inhibitor that affects kinase activity of both JNK1 and JNK2, was followed by a modest decrease of inflammation and paw swelling and an impressive inhibition of radiographic damage [21]. The influence of selective deletion of JNK2 was investigated in collagen-induced arthritis in mice. The severity of arthritis was even slightly increased, and histological evaluation showed a degree of synovial inflammation comparable to that in wt mice [25]. These latter findings suggest that selective inhibition of JNK2 is not effective to block destructive arthritis and raises the question whether JNK1 is a more promising target or effective inhibition of arthritis depends on the nonselective inhibition of JNK1 and JNK2. Our data extend this knowledge: the selective inhibition of JNK1 is not effective to block inflammation in TNF-driven arthritis. In fact, we show that even in the complete absence of JNK1, phosphorylation of JNK, although to a reduced amount, still occurs via engagement of JNK2. The latter is expressed in the synovial inflammatory tissue and its activation by TNF is sufficient to induce c-Jun transcription factor. Taken together, these findings from various experimental models of arthritis suggest that only the inhibition of both JNK isoforms, JNK1 and JNK2, may be a feasible approach to achieve a major blockade of synovitis and thus achieve therapeutic efficacy.

Development of local bone erosions in the joints affected by chronic arthritis depends on the presence of osteoclasts [13,26]. Interestingly, JNK1 plays an important role in osteoclastogenesis. Cells derived from *JNK1*^-/- ^mice revealed an impaired differentiation of osteoclasts *in vitro *[27]. Thus, targeting of JNK1 could have been considered as a feasible approach to protect from inflammatory joint damage. Surprisingly, however, the degree of bone erosions as well as numbers of osteoclasts was not affected by the lack of JNK1, indicating that TNF-driven osteoclastogensis is independent of JNK1 *in vivo*.

In fact, matrix metalloproteinases substantially contribute to irreversible degradation of collagen. The IL-1-dependent induction of matrix metalloproteinases in chondrocytes is regulated through complex pathways including MAPKs, AP-1, and nuclear factor κB transcription factors [28]. Interestingly, lack of JNK2 led to a modest but significant reduction of cartilage damage in collagen-induced arthritis [25]. Therefore, we hypothesized that a reduction of cartilage damage could be expected after knocking out JNK1 in hTNFtg mice. However, much as with synovial inflammation and bone loss, no significant protection of articular cartilage has been observed in *JNK1*^-/-^hTNFtg, suggesting that JNK1 does not seem to be important in TNF-mediated cartilage destruction.

## Conclusion

Taken together, these findings show that JNK1 is not essential for TNF-mediated joint disease. Specific inhibitors of JNK1 in mice probably work in conditions that are not primarily dependent on TNF-α. Moreover, an effective inhibition of synovitis and joint destruction may necessitate a combined blockade of the JNK isoforms or even additional MAPKs.

## Abbreviations

Ab = antibody; AP-1 = activator protein-1; H&E = hematoxylin and eosin; hTNFtg = human tumour necrosis factor transgenic; IL-1 = interleukin-1; JNK = c-Jun N-terminal kinase; MAPK = mitogen-activated protein kinase; PBS = phosphate-buffered saline; RA = rheumatoid arthritis; TNF = tumour necrosis factor; TRAP = tartrate-resistant acid phosphatase; wt = wild-type.

## Competing interests

The author(s) declare that they have no competing interests.

## Authors' contributions

MK carried out histological analyses and drafted the manuscript.

SH, EW, and GS conceived of the study and participated in its design and coordination.

KR carried out histological and statistical analyses.

RR participated in breeding of mice.

JD participated in the design of the study and breeding of mice.

GSt coordinated immunohistological analysis.

JS participated in the design of the study.

All authors read and approved the final manuscript.

## Figures and Tables

**Table 1:** Cellular composition (%) of cells infiltrating synovial tissue in two mouse strains with arthritis

Cells	hTNFtg	*JNK1*^-/-^hTNFtg
Macrophages	49.5 ± 7.8	50.5 ± 5.3
Granulocytes	17.7 ± 1.5	15.5 ± 2.5
T lymphocytes	5.0 ± 1.0	6.7 ± 0.6
B lymphocytes	6.3 ± 2.1	5.0 ± 1.8

Values are percentages (mean ± standard deviation) of positive cells among total cells in the synovial tissue. hTNFtg, human tumour necrosis factor transgenic; JNK, c-Jun N-terminal kinase.
